# Osteopontin Impairs Host Defense during Established Gram-Negative Sepsis Caused by *Burkholderia pseudomallei* (Melioidosis)

**DOI:** 10.1371/journal.pntd.0000806

**Published:** 2010-08-31

**Authors:** Gerritje J. W. van der Windt, W. Joost Wiersinga, Catharina W. Wieland, Ivo C. S. I. Tjia, Nicholas P. Day, Sharon J. Peacock, Sandrine Florquin, Tom van der Poll

**Affiliations:** 1 Center of Infection and Immunity Amsterdam (CINIMA), Academic Medical Center, Amsterdam, The Netherlands; 2 Center for Experimental and Molecular Medicine, Academic Medical Center, Amsterdam, The Netherlands; 3 Mahidol University, Bangkok, Thailand; 4 Centre for Clinical Vaccinology and Tropical Medicine, University of Oxford, Oxford, United Kingdom; 5 Department of Medicine, University of Cambridge, Cambridge, United Kingdom; 6 Department of Pathology, Academic Medical Center, Amsterdam, The Netherlands; University of Texas Medical Branch, United States of America

## Abstract

**Background:**

Melioidosis, caused by infection with *Burkholderia* (*B.*) *pseudomallei*, is a severe illness that is endemic in Southeast Asia. Osteopontin (OPN) is a phosphorylated glycoprotein that is involved in several immune responses including induction of T-helper 1 cytokines and recruitment of inflammatory cells.

**Methodology and Principal Findings:**

OPN levels were determined in plasma from 33 melioidosis patients and 31 healthy controls, and in wild-type (WT) mice intranasally infected with *B. pseudomallei*. OPN function was studied in experimental murine melioidosis using WT and OPN knockout (KO) mice. Plasma OPN levels were elevated in patients with severe melioidosis, even more so in patients who went on to die. In patients who recovered plasma OPN concentrations had decreased after treatment. In experimental melioidosis in mice plasma and pulmonary OPN levels were also increased. Whereas WT and OPN KO mice were indistinguishable during the first 24 hours after infection, after 72 hours OPN KO mice demonstrated reduced bacterial numbers in their lungs, diminished pulmonary tissue injury, especially due to less necrosis, and decreased neutrophil infiltration. Moreover, OPN KO mice displayed a delayed mortality as compared to WT mice. OPN deficiency did not influence the induction of proinflammatory cytokines.

**Conclusions:**

These data suggest that sustained production of OPN impairs host defense during established septic melioidosis.

## Author Summary

Melioidosis is a severe tropical disease caused by infection with the bacterium *Burkholderia* (*B.*) *pseudomallei*. In northeast Thailand infection with this bacterium is the major cause of community-acquired septicemia with a mortality rate up to 40%. Extending the knowledge on the mechanisms of host defense against *B. pseudomallei* infection would be helpful to improve treatment of this severe illness. Osteopontin (OPN) is a cytokine that is involved in several immune responses that occur during bacterial infection. In this study, we investigated levels of OPN in patients with melioidosis, and studied the function of OPN during experimental melioidosis in mice. We found that OPN concentrations were elevated in patients with severe melioidosis, and that high OPN concentrations are associated with poor outcome in patients with melioidosis. In experimental melioidosis in mice plasma and lung OPN levels were also increased. Moreover, mice with melioidosis that were deficient for OPN demonstrated reduced bacterial numbers in their lungs, diminished pulmonary tissue injury, and decreased neutrophil infiltration into the lungs during established melioidosis. Moreover, these mice displayed a delayed mortality as compared to control mice. In conclusion, sustained production of OPN impairs host defense during melioidosis.

## Introduction

Melioidosis is an important cause of severe sepsis in Southeast Asia and Northern Australia caused by the aerobic gram-negative soil-dwelling bacillus *Burkholderia* (*B.*) *pseudomallei* [1], [2]. Infection is thought to occur by cutaneous inoculation or inhalation. More than half of patients with melioidosis present with pneumonia associated with bacterial dissemination to distant organs [3], and all-cause mortality is as high as 50% in Northeast Thailand where the majority of reported cases occur [4].

*B. pseudomallei* is a facultative intracellular pathogen that multiplies in the host cell cytosol [5], [6]. Although the pathogenesis of melioidosis is still largely unknown, both innate and adaptive responses are important for an adequate host response [7]. Patients with severe melioidosis demonstrate elevated serum concentrations of several cytokines, including the T-helper (Th) 1 cytokines interferon (INF)-γ and interleukin (IL)-12 and IL-18 [8], [9]. Murine studies on the functional role of these cytokines and on tumor necrosis factor (TNF)-α during experimental melioidosis have shown enhanced mortality and bacterial outgrowth when one of these mediators was absent or inhibited, demonstrating the importance of these cytokines for host defense against *B. pseudomallei* [9]–[11]. Moreover, accumulating data show a vital role for neutrophils and macrophages in the host response to this pathogen [12]–[17].

Osteopontin (OPN) is a phosphorylated glycoprotein expressed by a broad range of tissues and cells that is involved in a number of physiological and pathological processes. OPN has been implicated in the regulation of inflammation; OPN acts as a chemotactic factor for T-cells, macrophages and neutrophils and modulates the function and differentiation of these inflammatory cells [18]–[23]. Several *in vitro* and *in vivo* studies indicate that OPN stimulates Th1 responses by inducing IL-12 and IFN-γ [24]–[27]. A recent report of elevated circulating OPN levels in patients with severe sepsis and septic shock further implicated this mediator in the pathogenesis of severe bacterial infection [28]. The contribution of OPN to the host response to bacterial infection has only been studied to a limited extent [29], [30]. Here, we sought to investigate the function of OPN in sepsis caused by melioidosis. For this we determined OPN plasma levels in patients with severe melioidosis and studied the role of OPN using an established model of murine melioidosis [17], [31].

## Methods

### Ethics statement

The patient study was approved by both the Ministry of Public Health, Royal Government of Thailand and the Oxford Tropical Research Ethics Committee, University of Oxford, England. We obtained written informed consent from all subjects before the study. The Animal Care and Use Committee of the University of Amsterdam approved all animal experiments.

### Patients

We included 33 individuals with sepsis caused by *B. pseudomallei* and 31 healthy controls in this study. Individuals were recruited prospectively at Sapprasithiprasong Hospital, Ubon Ratchathani, Thailand in 2004. Sepsis due to melioidosis was defined as culture positivity for *B. pseudomallei* from any clinical sample plus a systemic inflammatory response syndrome (SIRS). To meet the SIRS criteria patients had to have at least three of the following four criteria: a core temperature of ≥38°C or ≤36°C; a heart rate of ≥90 beats/min; a respiratory rate of ≥20 breaths/min or a PaCO_2_ of ≥32 mmHg or the use of mechanical ventilation for an acute respiratory process; a white-cell count of ≥12×10^9^/l or ≤4×10^9^/l or a differential count showing >10% immature neutrophils [32]. Study design and subjects have been described in detail elsewhere [33]. Blood samples for OPN measurements were drawn in heparin anticoagulated tubes in all subjects (once from controls and from patients within 36 hours after the initiation of antibiotic therapy and where possible at the end of intravenous treatment with antibiotics).

### Cell cultures

Stimulation of alveolar macrophages and respiratory epithelial cells was done as described previously [17]. In brief, the murine alveolar macrophage cell line MH-S (American Type Culture Collection; ATCC CRL-2019; Rockville, MD) was grown in RPMI 1640 (Gibco, Life Technologies, Rockville, MD) containing 2 mM l-glutamine, penicillin, streptomycin and 10% fetal calf serum, supplemented with 50 µM 2-βME (Sigma, Aldrich, St. Loius, MO). The murine transformed ATII respiratory epithelial cell line MLE-15 was generously provided by Jeffrey Whitsett (Cincinnati Children's Hospital Medical Center, Cincinnati) and was cultured in HITES medium (RPMI 1640 supplemented to 5 µg/ml insulin, 10 µg/ml transferrin, 30 nM sodium selenite, 10 nM hydrocortisone and 10 nM β-estradiol) supplemented with 2% FCS, penicillin and streptomycin. *In vitro* stimulation of cell-lines was conducted in 96-well plates (Greiner, Alphen aan de Rijn, the Netherlands) at a density of 5×10^5^ cells/ml. Following overnight culture at 37°C in 5% CO_2_, adherent cells were washed twice and then stimulated with heat-killed *B. pseudomallei* (strain 1026b, kindly provided by Dr. Don Woods, University of Calgary, Canada, multiplicity of infection 1∶10 and 1∶100) at 37°C in 5% CO_2_. Supernatants were taken after 4 hours of stimulation and stored at −20°C until assayed for OPN. Primary alveolar macrophages were harvested and stimulated as described previously [33], [34].

### Mice

Pathogen-free male WT C57BL/6 mice were purchased from Harlan Sprague Dawley Inc. (Horst, The Netherlands). OPN knockout (KO) mice, on a C57BL/6 genetic background, were obtained from the Jackson Laboratories (Bar Harbor, ME) and bred in the animal facility of the Academic Medical Center (Amsterdam, The Netherlands).

### Study design

Melioidosis was induced by intranasal inoculation of ∼10^3^ colony forming units (CFU) of *B. pseudomallei*, strain 1026b in 50 µl 0.9% NaCl, as described previously [17], [31]. Mice were sacrificed after 24 or 72 h (n = 8 per group), when blood was drawn into heparin containing tubes, and organs were removed aseptically and homogenized in 5 volumes of sterile 0.9% NaCl using a tissue homogenizer (Biospec Products, Bartlesville, OK). CFUs were determined from serial dilutions of organ homogenates and blood, plated on blood agar plates and incubated at 37°C at 5% CO_2_ for 16 h before colonies were counted. For survival studies mice (n = 14 per group) were monitored for 14 days after infection.

### Histology

Lungs and livers were harvested 24 and 72 hours after infection, fixed in 10% buffered formalin for 24 h, and embedded in paraffin. Hematoxilin and eosin stained slides were coded and scored from 0 (absent) to 4 (severe) by a pathologist blinded for groups. For lung the following parameters were scored: interstitial inflammation, endothelialitis, bronchitis, edema, necrosis and pleuritis. The total “lung inflammation score” was expressed as the sum of the scores for each parameter, the maximum being 24. Confluent (diffuse) inflammatory infiltrate was quantified separately and expressed as percentage of the lung surface. For liver abscess, necrosis and thrombus formation was scored.

### Assays

OPN, keratinocyte-derived cytokine (KC), macrophage inflammatory protein (MIP)–2, and LPS-induced CXC chemokine (LIX) were measured by ELISA (R&D Systems, Abingdon, UK). IL-12p70, IFN-γ, TNF-α, IL-6, IL-10 and monocyte chemoattractant protein (MCP)-1 were measured by cytometric bead array multiplex assay in accordance with the manufacturer's recommendations (BD Biosciences, San Jose, CA). Myeloperoxidase (MPO) was measured by ELISA (Hycult Biotechnology BV, Uden, The Netherlands). Aspartate aminotransferase (ASAT) and alanine aminotransferase (ALAT) were determined with commercially available kits (Sigma-Aldrich), using a Hitachi analyzer (Roche) according to the manufacturer's instructions.

### Statistical analysis

Values are expressed as mean ± standard error of the mean (SEM). Differences between groups were analyzed by the Mann-Whitney U test, preceded by Kruskal-Wallis analysis where appropriate. Paired patient samples were analyzed using a Wilcoxon signed rank test. Survival curves were compared by Kaplan-Meier analysis followed by log-rank test. These analyses were performed using GraphPad Prism version 4.0, GraphPad Software (San Diego, CA). Values of *P*<0.05 were considered statistically significant.

## Results

### Elevated plasma osteopontin levels in patients with culture proven melioidosis

**Figure 1:**
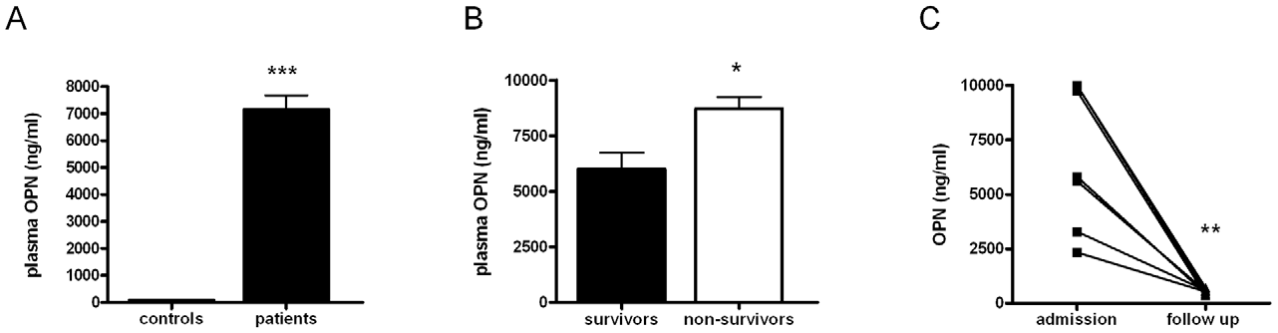
OPN plasma levels are elevated in patients with melioidosis and correlate with mortality. OPN plasma concentrations in (A) healthy controls versus melioidosis patients (n = 31 and n = 33, respectively), (B) survivors versus non-survivors (n = 19 and n = 14, respectively), and (C) patients at admission versus follow up sampling (n = 8). Data are expressed as means ± SEM, * *P*<0.05, ** *P*<0.01 and *** *P*<0.001.

To obtain a first insight into OPN expression during melioidosis, we measured OPN in plasma from 33 patients with septic melioidosis and from 31 healthy controls. The mortality rate in this cohort of patients was 44% [33]. OPN levels were approximately 70-fold higher in melioidosis patients than in healthy controls (figure 1A, *P*<0.001). On admission, patients who went on to die had higher OPN plasma levels than patients who survived (figure 1B, *P*<0.05). Furthermore, in eight patients from whom a second blood sample was obtained at the end of a two-week treatment period, plasma OPN levels had dramatically decreased as compared to levels measured on admission to the hospital (figure 1C, *P*<0.01).

### Osteopontin levels are increased during murine melioidosis

**Figure 2:**
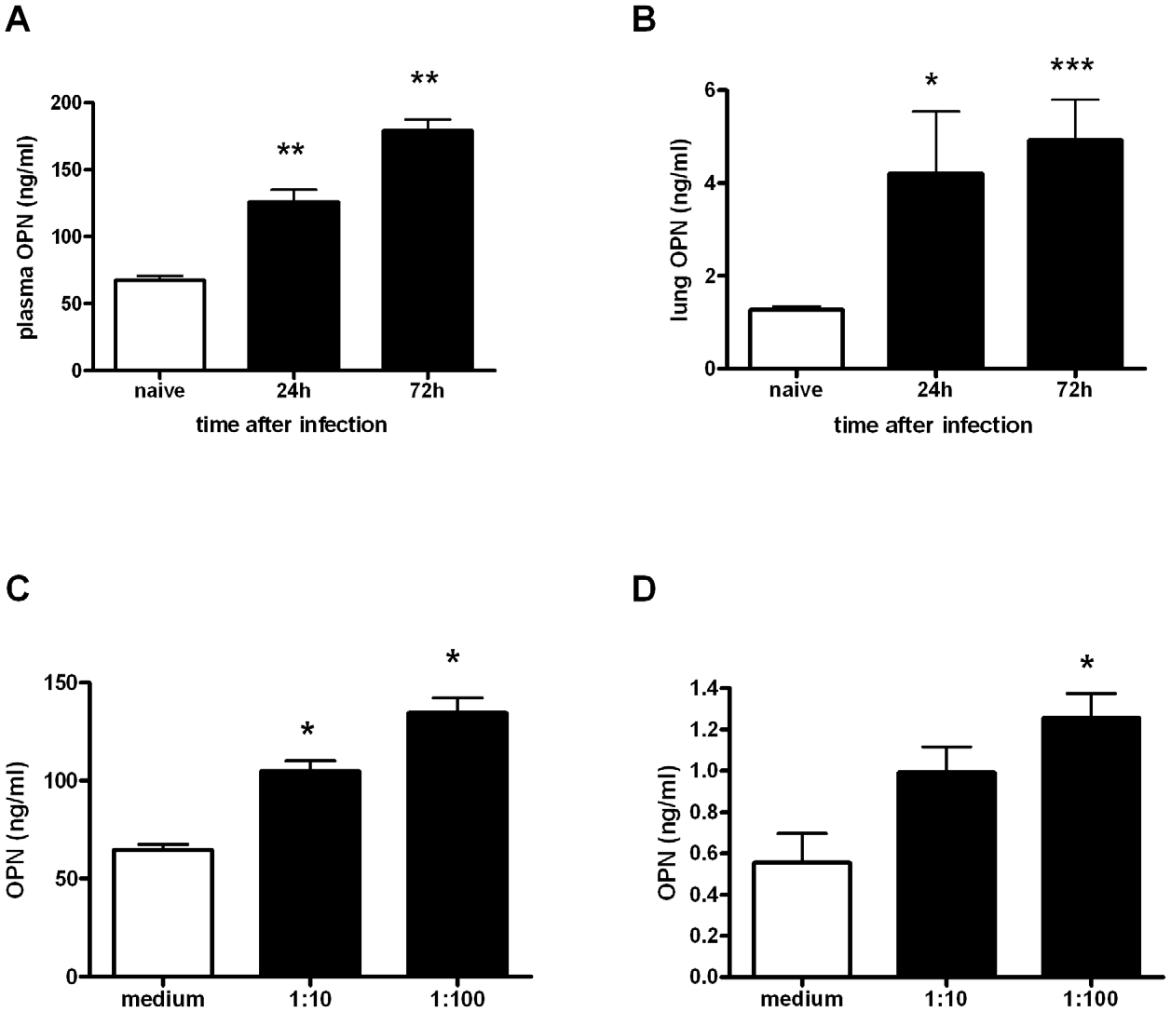
Experimental murine melioidosis results in elevated OPN concentrations in plasma and lungs. OPN concentrations in (A) plasma and (B) lung before, 24 and 72 h after intranasal infection with 10^3^ CFU of *B. pseudomallei*. Data are expressed as means ± SEM; n = 8 mice/group, * *P*<0.05, ** *P*<0.01 and *** *P*<0.001 as compared to t = 0. OPN concentrations in culture supernatants after incubation of (C) MH-S and (D) MLE-15 cells with medium or heat-killed *B. pseudomallei* (MOI 1∶10 and 1∶100) for 4 h. Data are expressed as means ± SEM; n = 4/group, * *P*<0.05 as compared to medium stimulation.

To determine whether OPN production is enhanced during melioidosis in mice, we intranasally infected WT mice with *B. pseudomallei* and measured OPN levels in plasma and whole lung homogenates (figure 2A–B). At 24 h after infection plasma OPN concentrations were significantly elevated compared to baseline (*P*<0.01) and were further increased at 72 h after infection (*P*<0.01, figure 2A). The same result was found for lung OPN levels (*P*<0.05 and *P*<0.001 for 24 and 72 h respectively, figure 2B). To determine whether OPN is released by pulmonary cells upon stimulation with *B. pseudomallei*, we incubated murine alveolar macrophage MHS and lung epithelial MLE-15 cells with medium or heat-killed *B. pseudomallei* (MOI 1∶10 and 1∶100) for 4 h. OPN release by both alveolar macrophages and epithelial cells was significantly enhanced upon *B. pseudomallei* stimulation in a dose dependent manner compared to the medium control (MHS: *P*<0.05 for both MOI, MLE-15: *P* = 0.05 for 1∶10 and *P*<0.05 for 1∶100, figure 2C–D).

### Osteopontin facilitates pulmonary bacterial growth

**Figure 3:**
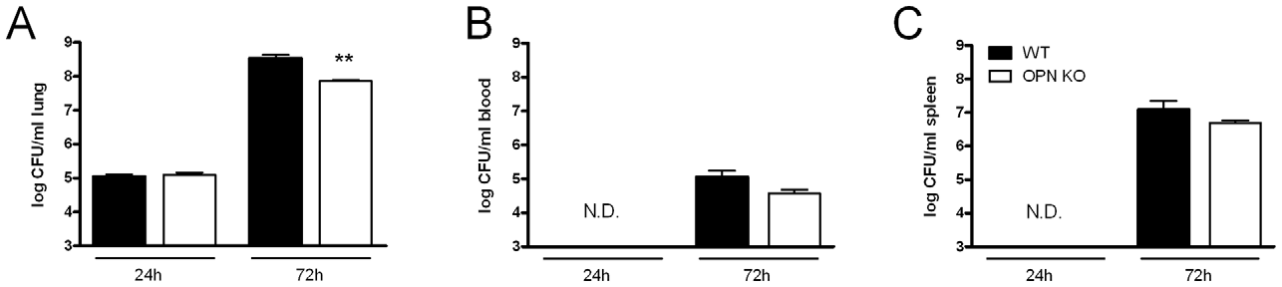
Decreased pulmonary bacterial growth in OPN KO mice. WT (black bars) and OPN KO (white bars) mice were infected intranasally with 10^3^ CFU of *B. pseudomallei* and bacterial loads were determined 24 and 72 h after infection in (A) lung, (B) blood, and (C) spleen. Data are expressed as means ± SEM; n = 8 mice/group, ** *P*<0.01 as compared to WT mice. N.D. means not detectable.

To determine whether OPN impacts on bacterial outgrowth during melioidosis, we determined bacterial loads in lungs from WT and OPN KO mice. At 24 h after infection bacterial outgrowth was similar in both groups. At 72 h after infection, however, bacterial loads were significantly decreased in lungs of OPN KO as compared to WT mice (figure 3A, *P*<0.01). To obtain insights into the dissemination of infection we determined bacterial loads in blood and spleen. Bacteria were not detectable in those distinct body sites at 24 h. After 72 h of infection, OPN KO mice had lower bacterial loads in blood and spleen than WT mice although the difference did not reach statistical significance (figure 3B–C).

### Osteopontin deficiency does not influence pulmonary cytokine concentrations

Since cytokines play an important role in host defense against *B. pseudomallei* [2] and OPN is known to act as a proinflammatory mediator able to induce cytokines including IFN-γ and IL-12 [24], [25], we measured proinflammatory cytokines (IFN-γ, IL-12p70, TNF-α, IL-6), an anti-inflammatory cytokine (IL-10) and chemokines (KC, MIP-2, LIX and MCP-1) in lung homogenates obtained 24 and 72 h after infection. With regard to chemokines we especially focused on neutrophil attracting proteins (i.e. KC, MIP-2 and LIX) considering that neutrophils are of utmost importance for host defense against melioidosis [12]. All measured cytokines and chemokines increased from 24 to 72 h after infection in both WT and OPN KO mice. Except for KC levels, which were significantly decreased in OPN KO lungs as compared to WT at both timepoints, no significant differences between the two mouse strains were found at either timepoint (table 1). In accordance with these *in vivo* data, primary alveolar macrophages harvested from uninfected WT and OPN KO mice released similar quantities of TNF-α, IL-6 and MIP-2, but reduced levels of KC (*P* = 0.05) upon incubation with *B. pseudomallei in vitro* (data not shown).

**Table 1:** Pulmonary cytokine concentrations.

	24h		72h	
ng/mL	WT	OPN KO	WT	OPN KO
**IL-12p70**	0.085±0.010	0.099±0.017	0.178±0.025	0.304±0.053
**IFN-γ**	0.067±0.008	0.129±0.074	0.213±0.019	0.235±0.013
**TNF-α**	0.531±85.80	0.369±0.140	6.389±0.738	4.595±0.860
**IL-6**	2.867±0.412	3.406±0.927	29.66±7.791	29.89±10.93
**IL-10**	0.226±0.028	0.259±0.006	0.604±0.106	0.607±0.088
**KC**	33.61±4.47	18.42±3.105*	262.2±26.85	166.8±26.31*
**MIP-2**	6.581±0.522	6.286±1.303	265.7±26.56	244.8±14.67
**LIX**	11.19±0.801	13.07±0.886	40.20±4.522	40.99±5.958
**MCP-1**	4.680±0.450	2.873±0.954	72.74±8.493	85.53±29.98

Proinflammatory cytokine (IL-12p70, IFN-**γ**, TNF-α and IL-6), anti-inflammatory IL-10 and chemokine (MIP-2, KC, LIX and MCP-1) levels in lung at 24 and 72 h after intranasaal *B. pseudomallei* infection in WT and OPN KO mice. Data are expressed as means ± SEM; n = 8 mice/group/time point. * *P*<0.05 vs. WT at the same time point.

### Osteopontin contributes to pulmonary inflammation

**Figure 4:**
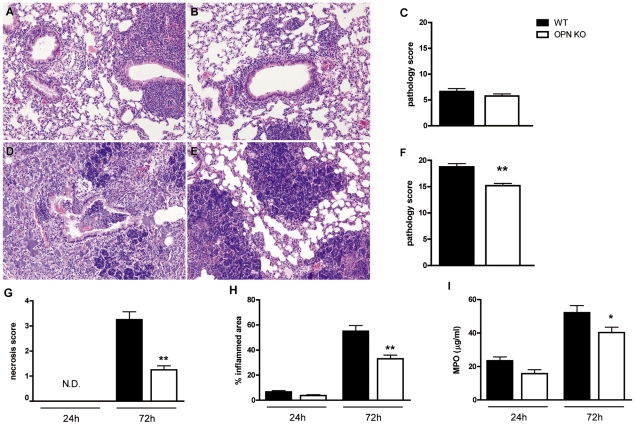
Reduced lung pathology in OPN KO mice. Representative lung pathology of WT (A, D) and OPN KO (B, E) mice 24 (A–C) and 72 (D–F) h after intranasal infection with 10^3^ CFU of *B. pseudomallei*. (G) Necrosis score and (H) the percentage of the inflamed area of the lung. (I) Myeloperoxidase levels in lung homogenates at 24 and 72 h after infection. The lung sections are representative for 8 mice per group per timepoint. H&E staining, original magnification 10×. Quantitative data are expressed as means ± SEM of 8 mice per group. * *P*<0.05 and ** *P*<0.01 as compared to WT.

To study the influence of OPN on pulmonary inflammation during melioidosis, lung histology slides obtained 24 and 72 h after infection were semi-quantitatively scored as described in the Methods section. In both WT and OPN KO mice pulmonary inflammation was characterized by significant interstitial inflammation, pleuritis, bronchitis, endothelialitis and edema, which increased from 24 to 72 h after infection. At the later time-point necrotic lesions could also be found. Lung inflammation scores were similar in both groups at 24 h after infection (figure 4A–C). At 72 h, however, lungs from OPN KO mice demonstrated significantly decreased inflammation scores as compared to WT, which was especially due to less necrotic tissue (*P*<0.01, figure 4D–G). In addition, OPN KO mice showed significantly reduced percentages of affected lung parenchyma (figure 4H). As neutrophils are the predominant infiltrating inflammatory cells during melioidosis [12], we also measured MPO in lungs from WT and OPN KO mice at 24 and 72 h after infection; MPO levels increased from 24 to 72 h in both groups. In accordance with the results on reduced percentages of affected lung, we found a trend towards reduced MPO levels in OPN KO lungs at 24 h (*P* = 0.10) and significantly diminished MPO levels in OPN KO lungs at 72 h after infection (figure 4I, *P*<0.05).

### Osteopontin does not influence hepatocellular injury during melioidosis

**Figure 5:**
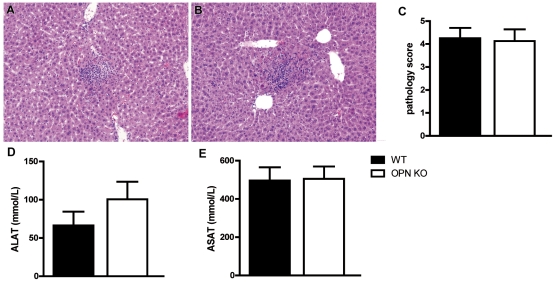
OPN does not influence hepatocellular injury. Representative liver pathology of WT (A) and OPN KO (B) mice 72 h after intranasal infection with 10^3^ CFU of *B. pseudomallei*. (C) Liver pathology scores (D) ALAT and (E) ASAT levels were similar at 72 h post infection. The liver sections are representative for 8 mice per group per timepoint. H&E staining, original magnification 10×. Quantitative data are expressed as means ± SEM of 8 mice per group.

Our model of melioidosis is associated with hepatocellular injury [9], [31]. To establish the role of OPN in this process we semi-quantitatively scored liver histology slides obtained from WT and OPN KO mice 24 or 72 h after infection. At 24 h post infection, liver histology was unremarkable in both groups (data not shown). At 72 h, both WT and OPN KO mice displayed abscesses, necrosis and thrombus formation (figure 5A and B); pathology scores were not different between groups (figure 5C). Consistent with this was the finding that plasma levels of ALAT and ASAT were similar in WT and OPN KO mice at this time point (figure 5D–E).

### Osteopontin impacts on mortality due to severe melioidosis

**Figure 6:**
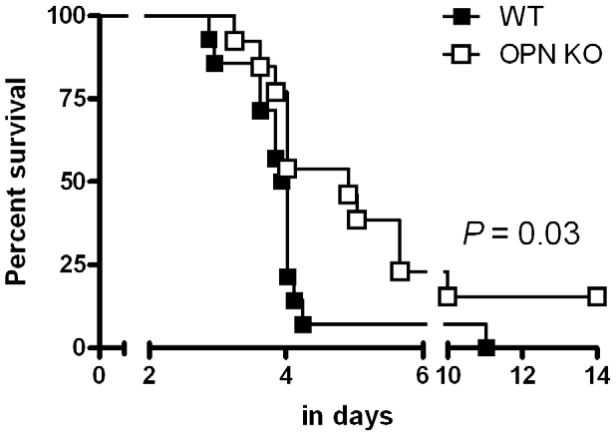
OPN KO mice show delayed mortality. Percentage survival of WT (closed symbols) and OPN KO (open symbols) mice after intranasal infection with 10^3^ CFU of *B. pseudomallei*. n = 14 per group. *P* value indicates the difference between groups.

To determine whether OPN affected mortality due to severe melioidosis, we followed WT and OPN KO mice for two weeks after *B. pseudomallei* infection. Consistent with reduced bacterial outgrowth and diminished pulmonary tissue injury in OPN KO mice, OPN deficiency had a positive effect on survival. Whereas WT mice showed a median survival time of 95 h, OPN KO mice had a median survival time of 118 h (*P*<0.05, figure 6).

## Discussion

In this study we sought to determine the role of OPN in the immune response to melioidosis. We demonstrate that plasma concentrations of OPN are elevated in patients with severe melioidosis and that high OPN levels on admission are correlated with mortality. In experimental murine melioidosis, OPN did not impact on the early host response; however, after 72 hours of infection OPN KO mice displayed reduced bacterial growth in lungs, accompanied by diminished pulmonary tissue injury and associated with delayed mortality. Taken together, these data suggest that enhanced production of OPN impairs host defense during established septic melioidosis.

Recently, serum levels of OPN were reported to be elevated in patients with sepsis caused by a mixture of pathogens and originating from various infectious sources [28]. The present study confirms and expands these data in a more homogenous cohort of patients with sepsis caused by a single pathogen, *B. pseudomallei*. Similar to a previous clinical study [28], we found that recovery from the acute phase was accompanied by a reduction in circulating OPN concentrations and that non-surviving patients had higher OPN levels on admission. Humans usually acquire melioidosis by inoculation through skin abrasions or inhalation, and pneumonia with bacterial dissemination to distant body sites is a common presentation of melioidosis [2], [3]. We therefore used a model of melioidosis in which mice were infected with *B. pseudomallei* via the airways. Using this model we confirmed increased release of OPN in the circulation and the lungs in mice and identified alveolar macrophages and respiratory epithelial cells as potential cellular sources for OPN upon infection with *B. pseudomallei*. Enhanced OPN production is not likely to be restricted to bacterial infections: elevated circulating OPN levels have previously been reported in patients with mycobacterial and fungal infections [35]–[38].

OPN has been shown to act as a chemo-attractant for several immune cells including macrophages, neutrophils and T cells [20]–[22], [39]. In particular, extracellular OPN is chemotactic towards neutrophils *in vitro* and *in vivo*, and the absence of intracellular OPN impairs migration speed and polarization upon *N*-formyl methionyl leucyl phenylalanine (fMLP) [22]. Considering that recruitment of neutrophils to the site of infection is critical for resistance against infection with *B. pseudomallei* [12], we anticipated that OPN KO mice would display a reduced resistance against experimentally induced melioidosis, especially since OPN may also contribute to a protective Th1 response during infection. However, whereas bacterial loads at 24 hours after infection were similar in OPN KO and WT mice, at 72 h post infection OPN deficiency was associated with an attenuated growth of *B. pseudomallei* in lungs and a tendency towards reduced dissemination. These findings were paralleled by an absence of an effect of endogenous OPN on the early recruitment of neutrophils into lung tissue, as reflected by histopathology and similar levels of MPO in whole lung homogenates from WT and OPN KO mice 24 h after infection. Of note, very recent data indicate that pneumonia induced by *Klebsiella pneumoniae*, like *B. pseudomallei* a gram-negative pathogen, results in a brisk rise in pulmonary OPN levels in WT mice and a diminished early neutrophil recruitment into the pulmonary compartment of OPN KO mice [30]. Together, these data suggest that the influence of endogenous OPN on neutrophil influx to the site of infection at least in part depends on the pathogen and its capacity to induce the local production of this mediator. At 72 h after infection, pulmonary OPN levels were still elevated, which together with the higher bacterial load might have contributed to the increased neutrophil numbers in lungs of WT mice; in addition, although early neutrophil recruitment is essential for resistance to *B. pseudomallei* infection [12], neutrophils can act as a “double-edged sword” and damage the architecture of lung tissue [40], [41], a mechanism that likely contributed to the enhanced lung injury in WT mice. Indeed, many studies have provided evidence for a crucial role of neutrophils in lung injury [42], [43]. Directly demonstrating such a detrimental role for neutrophils in melioidosis may be difficult, considering that neutrophil depletion results in much higher bacterial loads, thereby hampering direct comparison with the host with normal neutrophil counts [12]; in light of the fact that neutrophils are not the only cells contributing to lung injury it can be anticipated that the very high bacterial loads in neutrophil depleted mice cause lung injury by mechanisms that do not rely on neutrophils. On the other hand, the increased tissue necrosis in WT lungs may have facilitated the local growth of *B. pseudomallei* by providing a niche for this pathogen to escape killing by immune cells.

In previous studies, our laboratory demonstrated a link between an impaired *early* neutrophil influx (at 24 hours post infection) and a subsequently increased bacterial growth in MyD88 KO and urokinase type plasminogen activator receptor KO mice infected with *B. pseudomallei* [31], [44]. Our current data are not inconsistent with these earlier findings: early neutrophil influx (24 hours) did not differ between OPN KO and WT mice, whereas the fact that at 72 hours after infection lung MPO levels were lower in OPN KO mice likely was the consequence of the lower bacterial loads (providing a less potent chemotactic stimulus), possibly in combination with lower lung levels of the neutrophil chemoattractant KC.

In the last decade, numerous *in vitro* and *in vivo* studies have proposed OPN as an important player in Th1 responses [24]–[27], [45]. For example, OPN induces IL-12 release by murine peritoneal macrophages, whereas OPN prevents lipopolysaccharide induced production of the Th2 cytokine IL-10 [24]. Moreover, T-cell dependent IL-12 production by human peripheral blood mononuclear cells is enhanced by OPN, in part via its ability to regulate CD3-induced expression of IFN-γ and CD40L by T-cells [25]. In addition, OPN has been shown to contribute to Th1 responses *in vivo*, including experimental autoimmune encephalomyelitis, infection with herpes simplex virus-1 and with *Plasmodium chabaudi chabaudi* [24], [26], [27]. Considering that Th1 cytokines serve a protective role in the immune response to experimental melioidosis [9]–[11], we anticipated that OPN deficiency would result in an impaired host defense response due to an attenuated induction of protective Th1 cytokines. However, we found no significant differences in the levels of the Th1 cytokines IL-12 and IFN-γ between WT and OPN KO mice at any timepoint. In this respect, it should be noted that in a model of murine tuberculosis we also did not detect an effect of OPN on the Th1 response [29]. Similarly, normal immune responses of OPN KO mice have been reported during vaccinia virus, influenza virus, *Listeria monocytogenes* [46] and *Borrelia burgdorferi* infection [47], [48]. With regard to murine melioidosis it should be noted that natural killer cells, rather than T cells, are the main producers of IFN-γ [12]. In our model of melioidosis OPN did not impact on the release of other cytokines and chemokines with exception of KC, which was lower in OPN KO mice.

OPN deficiency did not impact on hepatocellular injury after infection with *B. pseudomallei*. OPN can be produced in the liver by various cell types, including Kupffer cells, macrophages, hepatic stellate and epithelial cells [49], [50]. Hepatic OPN has been implicated as a mediator of several forms of sterile liver injury, including T cell mediated hepatitis [51], acetaminophen-induced hepatic injury [52], nonalcoholic steatohepatitis [53] and carbon tetrachloride induced liver injury [54]. In contrast to these models, hepatocellular injury during experimental melioidosis is not caused by a direct toxic hepatic insult. Rather, liver damage is the result of dissemination of *B. pseudomallei* to distant organs and as a consequence thereof tissue damage. In a previous study, our laboratory demonstrated a link between the extent of bacterial dissemination and hepatocellular injury during murine melioidosis [33]. In the present study, bacterial loads in spleen and blood did not differ between OPN KO and WT mice. Together these data indicate that although OPN facilitates several forms of sterile hepatocellular injury, it does not play a role of importance in liver damage during septic melioidosis.

The latest sampling time point (72 hours) was chosen since the first deaths occurred shortly thereafter. Considering that WT mice displayed an accelerated mortality, comparisons between OPN and WT mice beyond 72 hours would be biased due to the fact that the most severely ill mice already had died.

OPN has been implicated in a variety of immune responses considered to contribute to an adequate host defense against invading pathogens. Here, we show that the production of OPN is enhanced during clinical and experimental melioidosis. Whereas endogenous OPN did not impact on the early host response during melioidosis in mice, its sustained release hampered the local control of the infection as reflected by reduced bacterial growth in lungs, less pulmonary inflammation and a delayed mortality in OPN KO mice. These data suggest that OPN plays a detrimental role in established sepsis caused by *B. pseudomallei* infection originating from the lung.

## References

[1] Cheng AC, Currie BJ (2005). Melioidosis: epidemiology, pathophysiology, and management.. Clin Microbiol Rev.

[2] Wiersinga WJ, van der Poll T, White NJ, Day NP, Peacock SJ (2006). Melioidosis: insights into the pathogenicity of Burkholderia pseudomallei.. Nat Rev Microbiol.

[3] Currie BJ (2003). Melioidosis: an important cause of pneumonia in residents of and travellers returned from endemic regions.. Eur Respir J.

[4] White NJ (2003). Melioidosis.. Lancet.

[5] Stevens MP, Wood MW, Taylor LA, Monaghan P, Hawes P (2002). An Inv/Mxi-Spa-like type III protein secretion system in Burkholderia pseudomallei modulates intracellular behaviour of the pathogen.. Mol Microbiol.

[6] Jones AL, Beveridge TJ, Woods DE (1996). Intracellular survival of Burkholderia pseudomallei.. Infect Immun.

[7] Wiersinga WJ, van der Poll T (2009). Immunity to Burkholderia pseudomallei.. Curr Opin Infect Dis.

[8] Lauw FN, Simpson AJ, Prins JM, Smith MD, Kurimoto M (1999). Elevated plasma concentrations of interferon (IFN)-gamma and the IFN-gamma-inducing cytokines interleukin (IL)-18, IL-12, and IL-15 in severe melioidosis.. J Infect Dis.

[9] Wiersinga WJ, Wieland CW, van der Windt GJ, de Boer A, Florquin S (2007). Endogenous interleukin-18 improves the early antimicrobial host response in severe melioidosis.. Infect Immun.

[10] Haque A, Easton A, Smith D, O'Garra A, Van Rooijen N (2006). Role of T cells in innate and adaptive immunity against murine Burkholderia pseudomallei infection.. J Infect Dis.

[11] Santanirand P, Harley VS, Dance DA, Drasar BS, Bancroft GJ (1999). Obligatory role of gamma interferon for host survival in a murine model of infection with Burkholderia pseudomallei.. Infect Immun.

[12] Easton A, Haque A, Chu K, Lukaszewski R, Bancroft GJ (2007). A critical role for neutrophils in resistance to experimental infection with Burkholderia pseudomallei.. J Infect Dis.

[13] Cheng AC, Limmathurotsakul D, Chierakul W, Getchalarat N, Wuthiekanun V (2007). A randomized controlled trial of granulocyte colony-stimulating factor for the treatment of severe sepsis due to melioidosis in Thailand.. Clin Infect Dis.

[14] Chanchamroen S, Kewcharoenwong C, Susaengrat W, Ato M, Lertmemongkolchai G (2009). Human polymorphonuclear neutrophil responses to Burkholderia pseudomallei in healthy and diabetic subjects.. Infect Immun.

[15] Breitbach K, Klocke S, Tschernig T, van Rooijen N, Baumann U (2006). Role of inducible nitric oxide synthase and NADPH oxidase in early control of Burkholderia pseudomallei infection in mice.. Infect Immun.

[16] Barnes JL, Ulett GC, Ketheesan N, Clair T, Summers PM (2001). Induction of multiple chemokine and colony-stimulating factor genes in experimental Burkholderia pseudomallei infection.. Immunol Cell Biol.

[17] Wiersinga WJ, de Vos AF, de Beer R, Wieland CW, Roelofs JJ (2008). Inflammation patterns induced by different Burkholderia species in mice.. Cell Microbiol.

[18] Scatena M, Liaw L, Giachelli CM (2007). Osteopontin: a multifunctional molecule regulating chronic inflammation and vascular disease.. Arterioscler Thromb Vasc Biol.

[19] Denhardt DT, Guo X (1993). Osteopontin: a protein with diverse functions.. Faseb J.

[20] Denhardt DT, Noda M, O'Regan AW, Pavlin D, Berman JS (2001). Osteopontin as a means to cope with environmental insults: regulation of inflammation, tissue remodeling, and cell survival.. J Clin Invest.

[21] Nystrom T, Duner P, Hultgardh-Nilsson A (2007). A constitutive endogenous osteopontin production is important for macrophage function and differentiation.. Exp Cell Res.

[22] Koh A, da Silva AP, Bansal AK, Bansal M, Sun C (2007). Role of osteopontin in neutrophil function.. Immunology.

[23] Wang KX, Denhardt DT (2008). Osteopontin: Role in immune regulation and stress responses.. Cytokine Growth Factor Rev.

[24] Ashkar S, Weber GF, Panoutsakopoulou V, Sanchirico ME, Jansson M (2000). Eta-1 (osteopontin): an early component of type-1 (cell-mediated) immunity.. Science.

[25] O'Regan AW, Hayden JM, Berman JS (2000). Osteopontin augments CD3-mediated interferon-gamma and CD40 ligand expression by T cells, which results in IL-12 production from peripheral blood mononuclear cells.. J Leukoc Biol.

[26] Chabas D, Baranzini SE, Mitchell D, Bernard CC, Rittling SR (2001). The influence of the proinflammatory cytokine, osteopontin, on autoimmune demyelinating disease.. Science.

[27] Maeno Y, Nakazawa S, Yamamoto N, Shinzato M, Nagashima S (2006). Osteopontin participates in Th1-mediated host resistance against nonlethal malaria parasite Plasmodium chabaudi chabaudi infection in mice.. Infect Immun.

[28] Vaschetto R, Nicola S, Olivieri C, Boggio E, Piccolella F (2008). Serum levels of osteopontin are increased in SIRS and sepsis.. Intensive Care Med.

[29] van der Windt GJ, Wieland CW, Wiersinga WJ, Florquin S, van der Poll T (2009). Osteopontin is not crucial to protective immunity during murine tuberculosis.. Immunology.

[30] van der Windt GJ, Hoogerwerf JJ, de Vos AF, Florquin S, van der Poll T (2010). Osteopontin promotes host defense during Klebsiella pneumoniae-induced pneumonia.. Eur Respir J published on-line (April 8).

[31] Wiersinga WJ, Wieland CW, Roelofs JJ, van der Poll T (2008). MyD88 dependent signaling contributes to protective host defense against Burkholderia pseudomallei.. PLoS ONE.

[32] Levy MM, Fink MP, Marshall JC, Abraham E, Angus D (2003). 2001 SCCM/ESICM/ACCP/ATS/SIS International Sepsis Definitions Conference.. Crit Care Med.

[33] Wiersinga WJ, Wieland CW, Dessing MC, Chantratita N, Cheng AC (2007). Toll-like receptor 2 impairs host defense in gram-negative sepsis caused by Burkholderia pseudomallei (Melioidosis).. PLoS Med.

[34] Wiersinga WJ, de Vos AF, Wieland CW, Leendertse M, Roelofs JJ (2008). CD14 impairs host defense against gram-negative sepsis caused by Burkholderia pseudomallei in mice.. J Infect Dis.

[35] Nau GJ, Chupp GL, Emile JF, Jouanguy E, Berman JS (2000). Osteopontin expression correlates with clinical outcome in patients with mycobacterial infection.. Am J Pathol.

[36] Inomata S, Shijubo N, Kon S, Maeda M, Yamada G (2005). Circulating interleukin-18 and osteopontin are useful to evaluate disease activity in patients with tuberculosis.. Cytokine.

[37] Koguchi Y, Kawakami K, Uezu K, Fukushima K, Kon S (2003). High plasma osteopontin level and its relationship with interleukin-12-mediated type 1 T helper cell response in tuberculosis.. Am J Respir Crit Care Med.

[38] Satie Nishikaku A, Scavone R, Fagnani Sanchez Molina R, Paulo Albe B, Da Silva Cunha C (2008). Osteopontin involvement in granuloma formation and in the severity of Paracoccidioides brasiliensis infection.. Med Mycol.

[39] O'Regan A, Berman JS (2000). Osteopontin: a key cytokine in cell-mediated and granulomatous inflammation.. Int J Exp Pathol.

[40] Zhang P, Summer WR, Bagby GJ, Nelson S (2000). Innate immunity and pulmonary host defense.. Immunol Rev.

[41] Craig A, Mai J, Cai S, Jeyaseelan S (2009). Neutrophil recruitment to the lungs during bacterial pneumonia.. Infect Immun.

[42] Abraham E (2003). Neutrophils and acute lung injury.. Crit Care Med.

[43] Zemans RL, Colgan SP, Downey GP (2009). Transepithelial migration of neutrophils: mechanisms and implications for acute lung injury.. Am J Respir Cell Mol Biol.

[44] Wiersinga WJ, Kager LM, Hovius JW, van der Windt GJ, de Vos AF (2010). Urokinase receptor is necessary for bacterial defense against pneumonia-derived septic melioidosis by facilitating phagocytosis.. J Immunol.

[45] Maeno Y, Nakazawa S, Dao le D, Van Tuan N, Giang ND (2006). Osteopontin is involved in Th1-mediated immunity against Plasmodium falciparum infection in a holoendemic malaria region in Vietnam.. Acta Trop.

[46] Abel B, Freigang S, Bachmann MF, Boschert U, Kopf M (2005). Osteopontin is not required for the development of Th1 responses and viral immunity.. J Immunol.

[47] Craig-Mylius K, Weber GF, Coburn J, Glickstein L (2005). Borrelia burgdorferi, an extracellular pathogen, circumvents osteopontin in inducing an inflammatory cytokine response.. J Leukoc Biol.

[48] Potter MR, Rittling SR, Denhardt DT, Roper RJ, Weis JH (2002). Role of osteopontin in murine Lyme arthritis and host defense against Borrelia burgdorferi.. Infect Immun.

[49] Kawashima R, Mochida S, Matsui A, YouLuTu ZY, Ishikawa K (1999). Expression of osteopontin in Kupffer cells and hepatic macrophages and Stellate cells in rat liver after carbon tetrachloride intoxication: a possible factor for macrophage migration into hepatic necrotic areas.. Biochem Biophys Res Commun.

[50] Banerjee A, Apte UM, Smith R, Ramaiah SK (2006). Higher neutrophil infiltration mediated by osteopontin is a likely contributing factor to the increased susceptibility of females to alcoholic liver disease.. J Pathol.

[51] Diao H, Kon S, Iwabuchi K, Kimura C, Morimoto J (2004). Osteopontin as a mediator of NKT cell function in T cell-mediated liver diseases.. Immunity.

[52] Welch KD, Reilly TP, Bourdi M, Hays T, Pise-Masison CA (2006). Genomic identification of potential risk factors during acetaminophen-induced liver disease in susceptible and resistant strains of mice.. Chem Res Toxicol.

[53] Sahai A, Malladi P, Melin-Aldana H, Green RM, Whitington PF (2004). Upregulation of osteopontin expression is involved in the development of nonalcoholic steatohepatitis in a dietary murine model.. Am J Physiol Gastrointest Liver Physiol.

[54] Morimoto J, Inobe M, Kimura C, Kon S, Diao H (2004). Osteopontin affects the persistence of beta-glucan-induced hepatic granuloma formation and tissue injury through two distinct mechanisms.. Int Immunol.

